# Lumbar vertebral osteomyelitis and psoas abscess caused by *Actinomyces israelii* after an operation under general anesthesia in a patient with end-stage renal disease: a case report

**DOI:** 10.1186/s13256-019-2261-y

**Published:** 2019-11-28

**Authors:** Yutaka Yamada, Chiharu Kinoshita, Hirokazu Nakagawa

**Affiliations:** 1 Department of General Internal Medicine, Kyoto Min-Iren Chuo Hospital, 16-1 Nishinokyo Kasugacho, Nakagyo Ward, Kyoto, 604-8453 Japan; 2 Department of Nephrology, Kyoto Min-Iren Chuo Hospital, Kyoto, Japan; 3 Department of Orthopedics, Kyoto Min-Iren Chuo Hospital, Kyoto, Japan

**Keywords:** *Actinomyces israelii*, Vertebral osteomyelitis, Psoas abscess, General anesthesia tracheal intubation, Nasogastric tube

## Abstract

**Background:**

Actinomycosis is a chronic, slowly progressive infection caused by the *Actinomyces* species. Lumbar vertebral involvement of *Actinomyces israelii* is extremely rare; this is the first case report of lumbar vertebral osteomyelitis and psoas abscess caused by *Actinomyces israelii* after an operation under general anesthesia.

**Case presentation:**

A 66-year-old Japanese man with end-stage renal disease was admitted to our hospital for an operation for cervical canal stenosis. After the operation under general anesthesia, during which tracheal intubation and nasogastric tube insertion were performed, he developed low back pain. During a second hospitalization, computed tomography revealed osteolysis of the lumbar endplates of L2 and L3, swelling of the intervertebral disk of L2/L3, and swelling of the left psoas major muscle. Percutaneous drainage of the intervertebral disc was performed, and the culture of the aspirate grew *Actinomyces israelii*. Based on the susceptibility, ampicillin was administered but his condition did not improve. We changed the antibiotics to ampicillin-sulbactam for coverage of unidentified oral commensals, and his symptoms and signs finally improved.

**Conclusion:**

Our patient’s long-term end-stage renal disease had made the oral and gastrointestinal mucosal barriers very fragile. Under these conditions, even mildly invasive procedures such as tracheal intubation and nasogastric tube insertion could be the cause of infectious complication by oral commensals, including *Actinomyces*.

## Background

Actinomycosis is a chronic, slowly progressive infection caused by the *Actinomyces* species, which are commensals of the oropharynx, gastrointestinal tract, and female genital tract [1]. *Actinomyces israelii* is the most common human pathogen. The organism is a filamentous, branching, Gram-positive, pleomorphic non-sporeforming bacillus. Cervicofacial actinomycosis is the most common form of the disease, followed by thoracic, abdominopelvic, and central nervous system diseases. Vertebral involvement of actinomycosis is rare and often secondary to an infection of contiguous tissue, such as cervicofacial or thoracic tissue [2, 3].

We experienced an extremely rare case of lumbar vertebral osteomyelitis and psoas abscess caused by *A. israelii* in a patient with end-stage renal disease after an operation under general anesthesia, during which a tracheal tube and a nasogastric tube were inserted.

## Case presentation

A 66-year-old Japanese man was referred to the orthopedic department of our hospital because of progressive weakness of his legs, difficulty in walking, and frequent falls. He also had bilateral weakness of upper extremities. Myelography performed 3 years before had already revealed severe cervical spinal canal stenosis. His symptoms and signs were considered to be caused by the deterioration of the cervical myelopathy. He was admitted for the surgical treatment of cervical spinal canal stenosis.

He had end-stage renal disease dependent on hemodialysis caused by lupus nephritis for 32 years. He had received laminoplasty of lumbar spine from L2 to L5 8 years before and laminectomy and posterior fixation of lower lumbar spine and sacrum from L4 to S2 2 years before. Seven years before he had also received pacemaker implantation and catheter ablation of atrioventricular node against refractory atrial fibrillation, which had persisted despite multiple catheter ablations of left atrium. Two years before he had received upper gastrointestinal endoscopy for routine screening, which showed ectopic gastric mucosa at cervical esophagus and nonspecific erosive gastritis. These findings looked the same as findings from previous examinations and a biopsy was not performed. His other medical history included peripheral arterial disease, secondary hyperparathyroidism, and hypothyroidism. His other surgical history included coronary artery bypass graft for severe left anterior descending artery stenosis, right total nephrectomy for renal cell carcinoma, multiple surgeries for bilateral carpal tunnel syndromes and amyloid arthropathies, and bilateral cataract surgeries. He had no remarkable history of dental and periodontal diseases. His medications were lanthanum carbonate, lansoprazole, warfarin, bisoprolol, cinacalcet, calcitriol, cilostazol, levothyroxine, sennoside, and vitamin E supplementation.

He received laminectomy of cervical spine from C4 to C6 and laminoplasty of C3 and C7 under general anesthesia on admission day 5. During the operation, a tracheal tube and a nasogastric tube were inserted. Cefazolin was administered intravenously as a perioperative prophylaxis. Weakness in his bilateral arms and legs was somewhat improved. However, his postoperative course was complicated with back pain and elevated C-reactive protein. On postoperative day (POD) 10, his back pain deteriorated, and ceftriaxone was administered. His back pain improved, and ceftriaxone was continued for 5 days. He was discharged on POD 34.

**Fig. 1:**
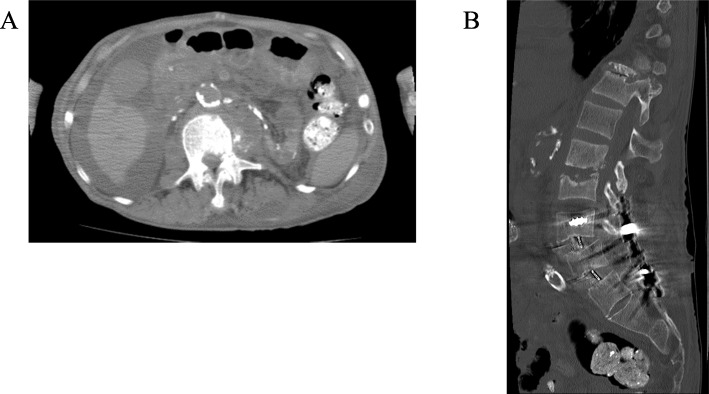
Computed tomography of lumbar spine and psoas abscess on day 5, the day after meropenem and vancomycin were initiated. **a** A coronal image showing low density area in the lateral part of left psoas major; **b** a sagittal image showing several bone pieces in the intervertebral area of L2/L3 and swelling of the disc. Although he had received two prior surgeries in his lower lumbosacral spine from L4 to S2, there was neither fracture nor abscess around the instruments

After discharge, his back pain gradually increased and he was unable to walk up stairs. He was readmitted to our hospital 12 days after discharge. Tramadol, acetaminophen, and loxoprofen were prescribed in the same manner as before the discharge. On day 4, his back pain worsened further, and a blood test showed white blood cell count of 22,400/μL, with neutrophils 85.5%, lymphocytes 9.5%, monocytes 4.0%, eosinophils 1.0%, and basophils 0.0%, and C-reactive protein 29.52 mg/dL. Computed tomography (CT) without contrast material revealed osteolysis of the lumbar endplates of L2 and L3, swelling of the intervertebral disk of L2/L3, and swelling of the left psoas major muscle (Fig. 1), without any finding indicative of surgical site infection of cervical spine. There was no sign of the involvement of the lower lumbar spine instruments. Magnetic resonance imaging was not performed since he had a cardiac pacemaker. Meropenem and vancomycin were initiated.

**Fig. 2:**
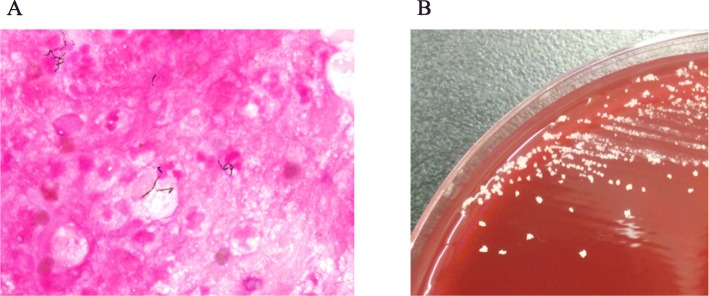
Gram stain and culture of the aspirate from L2/L3 intervertebral disk. **a** Gram stain showed multiple neutrophils and a few thin filamentous Gram-positive rods, and **b** small whitish colonies on sheep blood agar. The isolate was identified as *Actinomyces israelii*

On day 6, percutaneous drainage of the intervertebral disk of L2/L3 was performed. A Gram stain of the aspirate revealed thin filamentous Gram-positive rods, and an anaerobic culture showed *Actinomyces* species (Fig. 2). Our university laboratory was consulted for further identification, and 16S ribosomal ribonucleic acid (rRNA) gene sequence analysis identified this bacterium as *A. israelii*. The antibiotics susceptibilities of the strain are shown in Table 1. Meropenem and vancomycin were de-escalated to intravenously administered ampicillin based on a susceptibility test of *A. israelii* on day 16.

**Table 1:** Minimum inhibitory concentrations of *Actinomyces israelii* strain isolated from the aspirate

Antibiotics	MIC	Susceptibility
Penicillin	≤ 0.03	Susceptible
Ampicillin	≤ 0.03	Susceptible
Ampicillin-sulbactam	≤ 2	Susceptible
Amoxicillin-clavulanate	≤ 2	Susceptible
Piperacillin-tazobactam	≤ 16	Susceptible
Ceftizoxime	≤ 2	Susceptible
Cefmetazole	≤ 1	Susceptible
Imipenem	≤ 0.25	Susceptible
Meropenem	≤ 0.25	Susceptible
Clindamycin	0.25	Susceptible
Chloramphenicol	≤ 0.5	Susceptible

*MIC* minimum inhibitory concentration. Antibiotics susceptibilities were based on Clinical and Laboratory Standards Institute (CLSI) M100-S26

**Fig. 3:**
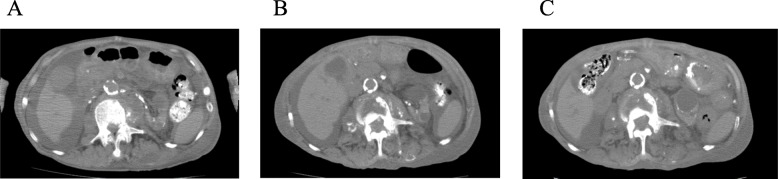
Computed tomography showed the gradual reduction of the left psoas abscess. **a** Day 5, the day after meropenem and vancomycin were initiated, **b** day 49, and **c** day 88

However, his back pain persisted, and a thin bloody exudate spilled over from his back on day 29. The culture of the exudate did not grow any organism. We thought that the causative organisms of his symptoms and signs were a mixture of oral bacterial flora, which could not be identified because of the antibiotic exposure before the aspiration. Intravenously administered ampicillin-sulbactam was substituted for ampicillin for coverage of beta lactamase-producing bacteria. Thereafter his back pain improved and the exudate diminished. Follow-up CT scans showed gradual reduction of the left psoas abscess (Fig. 3). On day 63, ampicillin-sulbactam was switched to orally administered amoxicillin-clavulanate. There was no recurrence of back pain and exudate. Three weeks later, he was transferred to a long-term care hospital, while amoxicillin-clavulanate was continued. He reportedly died of aspiration pneumonia 8 months after the transfer.

## Discussion

Actinomycosis is an endogenous infection. Risk factors of actinomycosis include poor oral hygiene, alcoholism, the use of intrauterine devices, and an immunocompromised condition such as diabetes mellitus and the use of immunosuppressive agents [3–7].

Vertebral involvement of *A. israelii* is very rare, especially in the lumbar spine. Only a few cases have been reported for lumbosacral *A. israelii* infection [8–12]. Although in one case the patient had poor oral hygiene and metastatic prostate cancer [8], in the other cases no obvious risk factor was reported [9–12].

In our case, a tracheal tube and a nasogastric tube were inserted during an operation on our patient’s cervical spine, which could have led to a mucosal lesion of his oral cavity and pharynx. Our patient had received hemodialysis for over 30 years. He had received an upper gastrointestinal endoscopy 2 years before presentation, which showed erosive gastritis. It has been reported that chronic kidney disease increases the permeability of the mucosa by loosening the tight junction of the epithelium [13]. In addition, he had taken lanthanum carbonate and proton pump inhibitor. A recent report [14] showed combined deposition of lanthanum and amyloid in the gastroduodenal mucosa of hemodialysis-dependent patients, which may cause fragility of the gastrointestinal barrier. The portal of *A. israelii* in this case could be the oral lesion or a more distal portion of the gastrointestinal tract, such as esophagus or stomach, although it could not be confirmed.

*A. israelii* is usually susceptible to penicillin G and aminopenicillins [4, 7]. However, in our case, our patient’s back pain and exudate were deteriorated after the de-escalation to ampicillin. It has been reported that *Actinomyces* are often isolated with other normal commensals, such as *Aggregatibacter actinomycetemcomitans*, *Eikenella corrodens*, *Capnocytophaga*, fusobacteria, *Bacteroides*, staphylococci, streptococci, or *Enterobacteriaceae*, depending on the site of infection [3, 15–17]. Oral anaerobes such as *Prevotella* and *Fusobacterium* species have a high rate of resistance to ampicillin, whereas they are almost always susceptible to ampicillin-sulbactam and amoxicillin-clavulanate [18, 19]. In a classical case series [20], two cases are reported in detail, who were initially treated with penicillin but eventually needed more broad-spectrum antibiotics because of concomitant other microbes. Since we initiated meropenem and vancomycin before the aspirate of the intervertebral disk was obtained for bacteriological studies, we probably could not identify the concomitant species which were susceptible to meropenem or vancomycin but resistant to ampicillin.

## Conclusion

Lumbar vertebral involvement of *Actinomyces* is extremely rare. Even mildly invasive procedures, such as tracheal intubation and nasogastric tube insertion, and predisposing gastrointestinal mucosal fragility could be the causes of infectious complication by oral commensals, including *Actinomyces*.

## Data Availability

All the data are available in Kyoto Min-Iren Chuo Hospital.

## Abbreviations

CT: Computed tomography
POD: Postoperative day
rRNA: Ribosomal ribonucleic acid
